# Genetic variation of the Alzheimer's disease risk gene CD33 modifies microglial immunometabolism

**DOI:** 10.1002/alz70855_107387

**Published:** 2025-12-25

**Authors:** Kirstin A Tamucci, Badri N. Vardarajan, Elizabeth M Bradshaw

**Affiliations:** ^1^ Columbia University, New York, NY, USA; ^2^ The Gertrude H. Sergievsky Center, College of Physicians and Surgeons, Columbia University, New York, NY, USA; ^3^ Department of Neurology, Columbia University, New York, NY, USA

## Abstract

**Background:**

The innate immune system has emerged as a central component of Alzheimer's disease (AD), with more than 75% of AD genetic loci being key innate immune genes. One such AD risk gene is CD33: a transmembrane, sialic‐acid binding protein that is specifically expressed by microglia in the brain. Previous studies suggest that CD33 regulates the inflammatory response by modifying microglial metabolism and oxidative phosphorylation. However, it remains unknown how CD33 genetic variation regulates microglial metabolism and how this contributes to AD pathogenesis.

**Method:**

To address this question, we generated human microglia‐like cells from primary monocytes with different CD33 genetic backgrounds and measured their respiratory bioenergetics and metabolic outcomes.

**Result:**

We found that the balance of ATP production via oxidative phosphorylation and glycolysis varies significantly with CD33 genotype. Compared to the AD‐risk genotype, those with the AD‐protective genotype had higher glycolytic activity but lower glucose uptake, specifically due to higher hexokinase activity, while no difference in lactate production or secretion was observed. To gain mechanistic insight, we used a mass spectrometry‐based approach to identify binding partners of the CD33 sialic acid binding domain, revealing a novel interaction between CD33 and the glucose transporter GLUT1. This result was further validated by PLA and co‐IP in a human monocytic cell line. Finally, using brain bulk RNA sequencing data from the ROSMAP cohort of AD patients and healthy elderly controls, we found CD33:GLUT1 gene interaction to be significantly associated with disease, underscoring the importance of this novel link between genetic, immune, and metabolic risk factors in AD.

**Conclusion:**

Together, these results provide insight on microglia‐specific metabolic changes due to variations in the AD‐associated risk gene CD33. This novel link between genetic, immune, and metabolic risk factors in AD has the potential to reveal new therapeutic and immunomodulatory strategies with which to reduce susceptibility to this neurodegenerative disease.

